# Variation on a theme; an overview of the Tn*916*/Tn*1545* family of mobile genetic elements in the oral and nasopharyngeal streptococci

**DOI:** 10.3389/fmicb.2014.00535

**Published:** 2014-10-20

**Authors:** Francesco Santoro, Morgana E. Vianna, Adam P. Roberts

**Affiliations:** ^1^Laboratory of Molecular Microbiology and Biotechnology, Department of Medical Biotechnologies, University of SienaSiena, Italy; ^2^Unit of Endodontology, UCL Eastman Dental Institute, University College LondonLondon, UK; ^3^Department of Microbial Diseases, UCL Eastman Dental Institute, University College LondonLondon, UK

**Keywords:** *Streptococcus*, conjugative transposon, antibiotic resistance, nasopharyngeal, oral cavity, transformation, conjugation, horizontal gene transfer

## Abstract

The oral and nasopharyngeal streptococci are a major part of the normal microbiota in humans. Most human associated streptococci are considered commensals, however, a small number of them are pathogenic, causing a wide range of diseases including oral infections such as dental caries and periodontitis and diseases at other body sites including sinusitis and endocarditis, and in the case of *Streptococcus pneumoniae*, meningitis. Both phenotypic and sequence based studies have shown that the human associated streptococci from the mouth and nasopharynx harbor a large number of antibiotic resistance genes and these are often located on mobile genetic elements (MGEs) known as conjugative transposons or integrative and conjugative elements of the Tn*916*/Tn*1545* family. These MGEs are responsible for the spread of the resistance genes between streptococci and also between streptococci and other bacteria. In this review we describe the resistances conferred by, and the genetic variations between the many different Tn*916*-like elements found in recent studies of oral and nasopharyngeal streptococci and show that Tn*916*-like elements are important mediators of antibiotic resistance genes within this genus. We will also discuss the role of the oral environment and how this is conducive to the transfer of these elements and discuss the contribution of both transformation and conjugation on the transfer and evolution of these elements in different streptococci.

## INTRODUCTION

The oral microbiota is one of the most diverse bacterial populations found in the human body (Topazian et al., 2002) with the total number of distinct taxonomic units found in the oral cavity in the 100s, if not the 1000s (Aas et al., 2005; Keijser et al., 2008; Wade, 2013), however, these are not all found in every mouth as individuals have distinct bacterial populations, and distinct bacterial populations are found in different habitats within each mouth (Eren et al., 2014 ). The vast majority of the oral inhabitants are commensal species and *Streptococcus* spp. are the most diverse and one of the most abundant genera present in saliva of both dentulous and edentulous individuals (Keijser et al., 2008; Ealla et al., 2013). The oral streptococci are pioneers in the colonization of the oral cavity soon after birth with the numbers and complexity of the oral bacterial species increasing gradually from exposure with microbial sources from the external environment and also following tooth eruption. *Streptococcus salivarius*, *S. mitis*, and *S. oralis* have been identified as the first and most dominant oral microbes in the oral cavities of newborns (Pearce et al., 1995).

Most *Streptococcus* spp. are non-pathogenic, forming part of the commensal human microbiome present in the mouth, intestine, and upper respiratory tract and they are also present on the skin. However, in the presence of host defense imbalance, many of these species can cause disease, such as dental caries and other infections (Topazian et al., 2002).

## DISEASES CAUSED BY ORAL STREPTOCOCCI

*Streptococcus* spp. are major residents of oral biofilms (dental plaque). Due to ecological shifts in the biofilm ecology a more lactic acid rich environment can result causing demineralization and destruction of the hard tissues of the teeth (e.g., enamel, dentin, and acellular cementum). *S. mutans* and *S. sanguinis* are strongly associated with early childhood caries, suggesting that the relative levels of these two microorganisms in the oral cavity play an important role in caries development in deciduous teeth (Hardie, 1992; Ge et al., 2008). The adhesion mechanisms of various *Streptococcus* spp. enable multiple intimate contacts and interactions between the bacterial cells and the host, which is crucial in biofilm formation, caries, and periodontal disease development. *In vitro* and *in vivo* studies have demonstrated direct roles for many streptococcal adhesins as colonization factors (Nobbs et al., 2009).

Periodontal disease corresponds to an inflammatory pathosis of bacterial origin resulting in the loss of tooth attachment and as a final consequence, the loss of the tooth (Nguyen and Martin, 2008). Although not the primary candidates for periodontal pathogens (Hardie, 1992), *S. mutans, S. sanguinis*, and *S. mitis* are the primary colonizers of the tooth surface and subsequent attachment of other bacteria results in a multispecies biofilm which can lead to inflammation (Ochiai et al., 1993). These species are also found in the colonized periodontal pockets (Edwardsson et al., 1999).

There is also evidence for the involvement of *Streptococcus* spp. in root canal infections with periapical disease, *S. gordonii*, *S. anginosus*, and *S. oralis* were the most frequently isolated streptococci from infected root canals, however, *S. mutans*, *S. intermedius*, *S. parasanguinis* have also been isolated (Chávez De Paz et al., 2003). Additionally other *Streptococcus* spp. have been detected in human infected root canals using checkerboard DNA–DNA hybridization including *S. mitis*, *S. intermedius*, and *S. constellatus* (Vianna et al., 2008). A clinical development of periapical disease is the dental abscess containing pus composed of dead host and bacterial cells. The causative microbiota is comprised of a mix of strict anaerobes in association with facultative anaerobes such as viridans and anginosus groups (Robertson and Smith, 2009). *S. anginosus*, *S. intermedius*, and *S. constellatus* have been identified from abscess cases in association with other species (Siqueira and Rôças, 2013).

A potential life-threatening complication of oral infections, involves the spreading of invasive microorganisms through connective tissue and fascial planes (cellulitis), brain, maxillary and cavernous sinus, eye, or mediastinum. The microbiology of the severe spreading odontogenic infection seems to differ from the more localized dental abscess with anginosus group streptococci and *Fusobacterium* spp. often isolated (Han and Kerschner, 2001).

Less specific, in terms of infected tissues is Noma, a gangrenous disease that affects maxillary of children with compromised immune function. This is most probably a polymicrobial infection, however, it has been demonstrated that *S. pyogenes* and *S. anginosus* (among other non-streptococcal species) are more abundant in the gingival microbiota of Noma patients with acute necrotizing gingivitis compared to healthy controls (Huyghe et al., 2013).

The spread of oral microorganisms by the blood circulation has been reported. Bacteraemia can occur as a result of tooth brushing (Lucas et al., 2008) and invasive procedures, however, these are usually transient in normal individuals and the exact definition of bacteraemia remains a topic of discussion (Bergstrom, 2009). However, in compromised hosts (e.g., patients with cancer, untreated diabetes or immunodeficiency) it can result in a generalized fatal infection (sepsis). The magnitude of bacteraemia of oral origin seems to be directly proportional to the degree of oral inflammation and infection (Bender et al., 1984), *S. viridans* (Heimdahl et al., 1990), *S. intermedius* and *S. sanguinis* (Debelian et al., 1995) have been isolated from blood samples. Bacteraemia is also considered a risk factor for the development of infective endocarditis, an infection of the heart valves (Starkebaum et al., 1977).

Antibiotics are administered as part of the clinical management of life-threatening infections and for the prevention of infective endocarditis in patients undergoing dental treatment, patients with prosthetic heart valves and/or patients with previous history of endocarditis and a cardiac transplant that develops a problem in a heart valve. The usual recommended antibiotics for these cases are: (1) oral amoxicillin, intravenous or intramuscular ampicillin, where patients are unable to take oral medication; (2) oral clindamycin, oral cephalexin, oral azithromycin or oral clarithromycin where patients are allergic to penicillin or ampicillin; (3) clindamycin or cefazolin (intravenous or intramuscular) where patients are allergic to penicillin or ampicillin and unable to take oral medication (Wilson et al., 2008). Although the use of systemic antibiotics is not a recommended routine to treat oral infections, its benefit is debatable when used in conjunction with non-surgical periodontal therapy (Keestra et al., 2014). The antibiotic resistance of isolates from dental abscess has increased over the years (Kuriyama et al., 2007) which is extremely concerning taking into consideration that general practitioners knowledge about antibiotics may not always be sufficient (e.g., Nabavizadeh et al., 2011) and its use can be indiscriminate.

## POLYMICROBIAL BIOFILMS OF THE ORAL CAVITY: A “GENE TRANSFER PRONE” ENVIRONMENT

The oral cavity, and to a lesser extent the nasopharyngeal cavity, are physicochemically complex environments, with many distinct habitats, in which different bacterial species coexist. Different bacteria are found in close contact on the mouth surfaces where they exist as polymicrobial biofilms. These are complex communities of bacteria adhering to a surface, embedded in a polymeric extracellular matrix consisting of polysaccharides, proteins and nucleic acids. Extracellular DNA (eDNA) is a key component (Whitchurch et al., 2002) of the biofilm matrix produced by many bacterial species (Roberts and Kreth, 2014). This eDNA has structural, nutritional, and informational value to the cells within the biofilm. Many of the species living in the oral cavity are naturally transformable, especially among streptococci, which are the most relevant genus of culturable microorganisms in the oral microflora (Cvitkovitch, 2001), these species can be transformed by eDNA present in the biofilm (e.g., Hannan et al., 2010). Beyond transformation, the close contact between bacteria in biofilms allows genetic exchange by means of conjugation, which requires cell-to-cell contact. Conjugation has been proven to happen in oral biofilms and in biofilms derived from different environments, with a frequency usually higher compared to conjugation between planktonically growing cells (Roberts et al., 1999, 2001a; Ready et al., 2006; Cook et al., 2011). There is a wealth of evidence suggesting that the oral biofilm environment is a suitable environment for extensive horizontal gene transfer and the readers are directed to recent reviews on this topic (Roberts and Mullany, 2006, 2010; Olsen et al., 2013; Roberts and Kreth, 2014).

## MOBILE GENETIC ELEMENTS – Tn*916*

Mobile genetic elements (MGEs) are regions of DNA that encode the necessary proteins in order to catalyze their own movement. MGEs are capable of movement within a bacterial cell, either between different sites within the same replicon or between replicons (e.g., from a chromosome to a plasmid). There are many different families of MGEs with the families being delineated by their structure or the type of recombinase responsible for their integration and excision from a replicon (Roberts et al., 2008). One of the types of elements which are often associated with the carriage of antibiotic resistance genes are the conjugative transposons, also known as integrative and conjugative elements (ICEs; Mullany et al., 2002; Roberts and Mullany, 2011). These MGEs are capable of both intracellular transfer between sites within a cell and intercellular conjugation using their self-encoded conjugation apparatus. There are different families of conjugative transposons, some of which seem to be confined to a particular species or genera of bacteria. Members of the Tn*916*/Tn*1545* family of conjugative transposons have, however, been found in, or introduced into, over 30 different genera of bacteria. The basic biology of Tn*916*/Tn*1545* like elements has been reviewed recently and readers are directed to these reviews (Roberts and Mullany, 2009, 2011; Ciric et al., 2011a).

Tn*916*, encoding *tet*(M) for the ribosomal protection protein Tet(M), was discovered and reported around 1980 (Franke and Clewell, 1981) when plasmid independent transfer of tetracycline resistance was demonstrated between strains of *Enterococcus faecalis*. Since then it, or variants of it (called here Tn*916*-like), has been found in many different organisms including important human pathogens such as *E. faecalis*, *Clostridium difficile*, *Staphylococcus aureus*, and *S. pneumoniae* (reviewed in Roberts and Mullany, 2011). Of these pathogens *S. pneumoniae* deserves particular attention in this review as its natural habitat is contiguous with the oral cavity and it is apparent that the influence of Tn*916*-like elements is similar in many respects to the activity and incidence of Tn*916*-like elements in the oral streptococci. In addition many of the elements found in the oral streptococci were initially discovered in *S. pneumoniae*.

## *Streptococcus pneumoniae*: DISEASE, COMPETENCE, AND ANTIBIOTIC RESISTANCE

*Streptococcus pneumoniae* is a member of the normal human nasopharyngeal microflora; it is present in a significant portion of the general population, with prevalence in children peaking at <5 years of age (Le Polain de Waroux et al., 2014). From the nasal cavity where it resides, *S. pneumoniae* can invade contiguous sites such as the ears and frontal sinus causing otitis media and sinusitis, which are mild but common diseases, and it can be aspirated in the lungs where it causes pneumonia. *S. pneumoniae* is also a causative agent of meningitis. It can reach meninges from the middle ear, the frontal sinus or from the bloodstream, which can be transiently invaded during pneumonia (Mook-Kanamori et al., 2011). The genome of *S. pneumoniae* shows high plasticity, with evidence of extensive horizontal gene transfer mediated by transformation and, to a lesser extent, conjugation and transduction. Natural genetic transformation was discovered in *S. pneumoniae* (Griffith, 1928) and since then has been intensively studied. In the process of transformation, a bacterium can internalize exogenous naked DNA and integrate it into its chromosome by homologous recombination. The acquisition, processing and integration of DNA are mediated by an array of proteins which are produced only transiently during the growth of the microorganism, in a phase called competence. The entrance of a pneumococcal population into the competence phase is regulated by a quorum sensing peptide called competence stimulating peptide (CSP), which is encoded by *com*C, synthesized as a 41 amino acid precursor, cleaved to 17 amino acids and secreted by the proteolytic ABC-transporter ComAB. CSP subsequently binds its receptor, which is a transmembrane histidine protein kinase coded by comD. Upon binding of CSP, ComD autophosphorylates, and donates the phosphoryl group to its cognate ComE response regulator (Martin et al., 2013). The phosphorylated ComE is able to bind to the promoter region of the early competence genes and activate their transcription: the activated operons include *com*AB and *com*CDE in an auto-induction loop (Peterson et al., 2004). The induction of competence by CSP is also essential for *S. pneumoniae* biofilm formation, suggesting that the two processes are intertwined and that competence has a deep effect on the cellular physiology (Oggioni et al., 2006). Antibiotic treatment has been demonstrated to induce competence in *S. pneumoniae*, the bacterium is therefore able to modify its genetic milieu and to acquire new phenotypes which might help its survival under stress conditions (Prudhomme et al., 2006). DNA can be acquired by transformation from co-colonizing pneumococci as well as from other commensal bacteria, which might be present in the same ecological niche, given enough homology between the transforming and recipient DNAs. Among the new phenotypes acquired by transformation there is increased resistance to antibiotics such as penicillin and cotrimoxazole (Chewapreecha et al., 2014).

Antibiotic resistance in *S. pneumoniae* is becoming more and more common, but has been known for a long time. Increased MIC for penicillin in a clinical strain of *S. pneumoniae* was first detected in 1967 in Australia (Hansman and Bullen, 1967), since then isolates with decreased susceptibility to penicillin have arisen and spread worldwide. An international network (Pneumococcal Molecular Epidemiology Network, PMEN) was established in 1997 in order to characterize and classify the international spread antibiotic resistant pneumococcal clones (McGee et al., 2001). The first identified clone is the Spain23F-1, also known as PMEN-1, resistant to penicillin, tetracycline, chloramphenicol, and cotrimoxazole.

In *S. pneumoniae*, resistance to tetracycline is often due to Tn*916*-like MGEs, which usually carry the *tet*(M) gene. Resistance to macrolides can be due to *erm* genes, coding for rRNA modifying methyltransferase, or to *mef*/*msr* genes which code for an eﬄux pump. The *erm* genes usually confer the MLS_B_ phenotype, resistance to macrolides, lincosamides and streptogramin B with high MIC values (>64 mg/L for erythromycin), while *mef*/*msr* genes confer the M phenotype, resistance to macrolides albeit with low MIC values (1–16 mg/L for erythromycin). In *S. pneumoniae*, both classes of resistance genes are carried by Tn*916*-like elements.

## Tn*916* VARIANTS DETECTED IN *S. pneumoniae* GENOMES

Many Tn*916*-family elements were originally detected and sequenced in clinical strains of *S. pneumoniae*. Eleven Tn*916*-like elements are present in 10 out of 25 complete pneumococcal genome sequences present in GenBank (as to 20th May 2014); strain CGSP14 harbors two different Tn*916*-like CTns (**Table 1**). In five out of 11 of these genomes, Tn*916*-like elements are integrated into a larger composite Tn*5253* family or ICESp23FST81 family element (see below), while in the remaining six cases they are present as individual elements. Nine out of the 11 Tn*916*-like elements are in fact composite elements themselves, as there are insertions of other DNA within the Tn*916* sequence backbone, two of these elements are also presumed defective, as they lack some of the Tn*916* sequences. The DNA sequences that can be found inserted within the Tn*916* backbone are: (i) Tn*917*, 5,089 bp, carrying the *erm*(B) gene, (ii) the “MEGA” element (macrolide eﬄux genetic assembly), 5,511 bp, carrying *mef*(E)/*msr*(D), (iii) the MAS (macrolide–aminoglycoside–streptothricin) element, 4,225 bp, carrying *erm*(B), *aadE*, *sat4*, and *aphA*-3 genes, and (iv) a 2,849 bp fragment containing *erm*(B). It is worth noting that the 2,849 bp *erm*(B) fragment derives from a 5,288 bp omega element (GenBank accession no. FR671418) which carries two copies of an omega repressor encoding gene and the *aphA*-3 gene (for a diagram of these elements and their location within Tn*916* readers a referred to Ciric et al., 2011a). The deletion of the *aphA*-3 fragment happens through a recombination event between the omega-encoding genes, and results in the fusion of the 3′ end of Tn*916 orf20* with the omega repressor coding sequence (Croucher et al., 2011).

**Table 1:** Complete pneumococcal genomes containing Tn*916*-family elements.

Strain (Acc. No.)	Element	Antibiotic resistance gene(s)	Insertions^a^ (bp)	Nucleotide of insertion^b^	Larger element (yes/no, family)
ATCC700669 (FM211187)	Tn*916*-like	*tet*(M)	None	–	Yes, ICE*Sp*23FST81
P1031 (CP000920)	Tn*916*-like	*tet*(M)	None	–	Yes, Tn*5253*
CGSP14 (CP001033)	Defective Tn*916*-like	*erm*(B) (two copies), *sat4*, *aphA-3*	MAS + Tn*917* (9339)	3847	Yes, ICE*Sp*23FST81
	Tn*3872*-like	*tet*(M), *erm*(B)	Tn*917* (5089)	14518	No
Taiwan19F-14 (CP000921)	Tn*2009*-like	*tet*(M), *mef*(E)/*msr*(D)	MEGA (5509)	14163	No
ST556 (CP003357)	Tn*2009*-like	*tet*(M), *mef*(E)/*msr*(D)	MEGA (5509)	14163	No
Hungary19A-6 (CP000936)	Tn*6002*-like	*tet*(M), *erm*(B)	*erm*(B) element (2849)	3840	No
670-6B (CP002176)	Tn*6002*-like	*tet*(M), *erm*(B)	*erm*(B) element (2849)	3840	Yes, Tn*5253*
A026 (CP006844)	Tn*2010*-like	*tet*(M), *erm*(B)	*erm*(B) element (2849)	3840	No
		*mef*(E)/*msr*(D)	MEGA (5509)	14163	
TCH8431/19A (CP001993)	Tn*2010*-like	*tet*(M), *erm*(B)	*erm*(B) element (2849)	3840	No
		*mef*(E)/*msr*(D)	MEGA (5509)	14163	
G54 (CP001015)	Defective Tn*6002*-like	*tet*(M), *erm*(B)	*erm*(B) element (2849)	3840	Yes, ICE*Sp*23FST81 (defective)

*^a^Compared to Tn*916* backbone.*

*^b^With reference to Tn*916* sequence (Acc. No. U09422).*

As reported in **Table 1**, the Tn*916*-like elements present in pneumococcal complete genome strains are: (i) Tn*3872*, a Tn*916* carrying Tn*917*, present in one strain (McDougal et al., 1998), (ii) Tn*6002* carrying the 2,849 bp *erm*(B) fragment, present in two strains (Warburton et al., 2007), (iii) Tn*2009*, carrying “MEGA,” in two strains (Del Grosso et al., 2004), and (iv) Tn*2010* carrying both “MEGA” and the 2,849 bp *erm*(B) fragment, in two strains (Del Grosso et al., 2006). Two more strains carry Tn*916*-like elements: one has a defective version of Tn*6002*, with a deletion from the end of *tet*(M) to the end of the element, and another resembles the structure of SpnRierm(B) (GenBank accession no. AM490850, Cochetti et al., 2007), where the MAS element is fused to Tn*917* replacing the portion from *orf19* to *tet*(M).

Tn*6003* and Tn*1545* are other Tn*916*-family elements which were first detected in *S. pneumoniae* and both of them contain the MAS element and the *erm*(B) fragment, the latter also has the insertion of a transposase at another site (Cochetti et al., 2008). It has recently been shown that the MAS element is able to excise and circularize using a 1,166 bp direct repeat containing *erm*(B), thus turning Tn*6003* into Tn*6002* (Palmieri et al., 2012).

A few epidemiological studies have reported the frequencies of the different elements in clinical strains of *S. pneumoniae*. A Chinese study performed on 328 invasive isolates from children <5 years in 2005 reported that 31% of the isolates were macrolide resistant, and among these, 74% were also tetracycline resistant (Xu et al., 2010). Investigation of the genetic elements present in the tetracycline and macrolide resistant strains was performed by PCR detection of integrase, excisionase, transposase, and resistance genes carried by Tn*916*-like elements, with subsequent reverse blot hybridization of the PCR product with specific probes and assignment to a category of genetic elements based on hybridization results. The study might have therefore underestimated the presence of new Tn*916* variants. Data analysis revealed that 49 strains carried Tn*6002*, 15 strains carried Tn*3872* and three carried Tn*1545*/Tn*6003*; variants of Tn*6002* and Tn*3872* lacking the *tet*(M) gene were also detected. A later study performed in China in 2010 on 135 macrolide resistant pneumococci from nasal swabs of children, confirmed the high prevalence of Tn*6002*-like elements (56.3% of the isolates), followed by Tn*2010* (28.9%), Tn*1545*/Tn*6003* (5.9%), and Tn*3872* (5.2%; Zhou et al., 2012). Tn*2010* was first detected in an Italian isolate (Del Grosso et al., 2006) and has later been completely sequenced (Li et al., 2011). In most strains analyzed, it was found to be inserted between bases 1,731,928 and 1,743,232 of the R6 genome (GenBank accession no. NC_003098), flanked at both ends by short sequence stretches (24 and 66 nucleotides) that did not show homology with the R6 genome nor with the ends of Tn*916*, in one strain it has also been found inserted between nucleotides 1,820,138 and 1,820,139 of the R6 genome, with only a 7 bp stretch at the left end (Li et al., 2011).

A PCR study performed on multidrug resistant pneumococcal strains colonizing children in Venezuela reported a high prevalence of Tn*3872* (44% of isolates) and the presence of Tn*6002* (10%), Tn*2009* (10%), Tn*2010* (4%), and an element related to SpnRierm(B) (only one isolate; Quintero et al., 2011). The elements were detected in strains of different serotypes, suggesting horizontal gene transfer of the resistance determinants, Tn*916*-family element are therefore widespread among multidrug resistant pneumococcal clinical isolates. Two studies on macrolide resistant pneumococci and their association with the presence of Tn*916* family of genetic elements were also performed in Spain (Calatayud et al., 2007, 2010), both of them reported the high prevalence of Tn*6002*-like elements (about 50% of macrolide resistant strains examined), a significant prevalence of Tn*3872* (about 25% of the strains) with a lower prevalence of the other elements. Interestingly, both the studies detected a number of strains (about 16%) harboring *erm*(B), *tet*(M), and *tndX*, suggesting the presence of an ICESp*1116* related element (GenBank accession no. HE802677; Brenciani et al., 2012). This element was originally described in *S. pyogenes* as a defective Tn*5397* with an *erm*(B) gene and an Insertion Sequence encoded transposase disrupting *tet*(M), with a recombination module including *tndX*, a large serine-recombinase (Wang and Mullany, 2000; Roberts et al., 2001b) and *orf45* coding for a DDE transposase.

## COMPOSITE ELEMENTS CARRYING Tn*916*-FAMILY ELEMENTS IN *S. pneumoniae*

It is well known that Tn*916* is a promiscuous element, as it can integrate at different sites not only into the host chromosome, but also into plasmids and into other larger ICEs. In *S. pneumoniae* at least two families of composite elements have integrated copies of Tn*916*; these are the Tn*5253* family and ICESp23FST81 family. The prototype elements are large (64.5 and 81 kbps respectively), share some sequence homology but have two different recombination modules and consequently different, specific integration sites in the *S. pneumoniae* chromosome. The insertion of Tn*5253* into *S. pneumoniae* chromosome leads the duplication of an 83 bp target site located between *spr1042* and the 5′ end of *spr1043* (*rbg*A; GenBank EU351020), while ICESpn23FST81 is inserted at the 3′ end of *spr1211* (*rpl*L), with its insertion causing a duplication of 16 bp (Croucher et al., 2009; insertion sites refer positions in the R6 genome). Both integration sites lie at one end of a pneumococcal conserved gene, whose coding sequence is not disrupted by the insertion of the element, this site selection strategy guarantees that the element always has a “safe site” to integrate in. These larger ICEs show a typical modular structure in which Tn*916*-like elements are inserted. The insertion site of Tn*916* within the larger element is variable, for instance in strains CGSP14 (GenBank CP001033), ATCC700619 (GenBank FM211187), and P1031 (GenBank CP000920) it integrates downstream Tn*5253 orf8* homolog; in strains 670-6B (GenBank CP002176) and G54 (GenBank CP001015) it integrates in a site not present in Tn*5253*, while in Tn*5253* it is integrated downstream *orf20*, coding for a truncated transposase of the IS*110* family. The presence of Tn*5253*-family and ICESp23FST81-family elements has been investigated in clinical isolates of *S. pneumoniae* and proven to be frequent, especially among multidrug resistant strains (Henderson-Begg et al., 2009; Mingoia et al., 2011); moreover ICESp23FST81-family elements are a distinctive feature of the isolates belonging to the multidrug resistant clone PMEN-1 (Croucher et al., 2011).

## BIOLOGY OF Tn*916*-LIKE ELEMENTS IN *S. pneumoniae*: IS THERE ANY EVIDENCE FOR CONJUGATION?

Tn*916* has limited requirements for target site selection in that it recognizes an A:T rich region (Scott et al., 1988, 1994; Mullany et al., 2012), consequently its host range is very broad (Bertram et al., 1991). However, it has been reported that Tn*916* integration can also occur at some specific or preferred sites, as in *Clostridium difficile* strain CD37 (Mullany et al., 1991; Wang et al., 2000) or in the enterococcal plasmid pAD1 (Jaworski and Clewell, 1994). In *S. pneumoniae* sequenced genomes, the insertions of Tn*916*-family elements can be mapped at a few hotspots (Santoro et al., 2010), suggesting a target site specificity. On the other hand, transconjugants generated in the lab harboring Tn*5251* (the Tn*916* component of the composite Tn*5253* element) seem to have multiple insertions at A:T rich sites within the chromosome (Santoro et al., 2010) and transconjugants generated in a serotype 3 *S. pneumoniae* strain, with *E. faecalis* as a Tn*916* donor, harbored multiple and widespread insertions (Watson and Musher, 1990). The actual functionality of Tn*916*-family elements in *S. pneumoniae* has been investigated in very few studies. Tn*3872*, originally described in 1998, was not capable of conjugal transfer to the standard pneumococcal recipient R6 (McDougal et al., 1998), this was later confirmed by Cochetti et al. (2007) who also showed that Tn*6002* and Tn*6003* are able to conjugate, albeit at low frequency and in a recipient dependent manner (transfer frequency of 1.1 × 10^-8^ to *S. pyogenes* 12RF and of 1.7 × 10^-7^ to *E. faecalis* JH2-2 CFU transconjugants/CFU donors, respectively). The “MEGA” carrying elements Tn*2009* and Tn*2010* are not conjugative using pneumococcal recipients and selection for either tetracycline or erythromycin resistance (transfer frequency<10^-9^ per donor cell), while both elements are transferable by transformation to standard pneumococcal recipients (Del Grosso et al., 2006). The first step in the conjugal transfer of Tn*916* family of genetic elements is the excision from the chromosome with formation of a circular intermediate (CI). A CI has been detected in strains carrying Tn*2010* (Zhou et al., 2014), indicating that XisTn and IntTn are probably functional, therefore the conjugal transfer may either happen at frequencies below the detection threshold or be impaired because of the insertion of “MEGA” within the regulatory region. The insertion of the *erm*(B) element in the conjugal transfer region does not seem to be detrimental for conjugation, as Tn*1545*, Tn*6002*, and Tn*6003* are conjugative. CIs have also been detected for Tn*5251* which is integrated in the composite element Tn*5253* (Provvedi et al., 1996), subsequently the autonomous transfer of Tn*5251* has been demonstrated, even if, again, at low frequencies and in a recipient dependent manner (Santoro et al., 2010). The fact that Tn*916*-family elements in *S. pneumoniae* tend to be either inserted in composite elements or at the same hotspots, suggests that their autonomous conjugation *in vivo* might not be frequent, even if there is evidence that this event is possible, and that they may rely on the conjugation machinery of larger elements such as ICESp23FST81 or Tn*5253* which are able to transfer at frequencies about three orders of magnitude higher (Iannelli et al., 2014).

## TRANSFORMABILITY OF *S. pneumoniae* WITH RESPECT TO CONJUGATIVE TRANSPOSONS: FROM PIONEERING STUDIES TO THE GENOMIC ERA

Tn*5253* had originally been identified as a chromosomal genetic element bearing resistance to chloramphenicol and tetracycline in the clinical strain BM6001. Work performed in the late seventies in the laboratories of Walter Guild investigated the transformation properties of pneumococcal lysates containing Tn*5253* (Shoemaker et al., 1979). First, isogenic strains with or without Tn*5253* were constructed, and then DNA contained in the lysates was used in transformation experiments. Different shearing treatments of donor DNA were used to infer the length of the heterologous insertions, the whole Tn*5253* was predicted to be longer than 30 kb and the *cat* containing fragment between 4 and 8 kb, which is consistent with the actual sizes; 64,528 and 7,627 bp, respectively. Transformation has been used to study the transferability of Tn*916* elements which could not be transferred by conjugation, the transformation frequencies of Tn*2009* and Tn*2010* have been shown to be 4 × 10^-7^ and 3 × 10^-7^ CFU/ml of recipient, respectively (Del Grosso et al., 2004). Whole genome sequencing of three Tn*2010* bearing transformants showed that multiple SNPs had been acquired together with the element; moreover the presence of the element did not involve any fitness cost (Zhou et al., 2014). A transforming pneumococcal DNA containing a “MEGA” carrying Tn*916*-like has been used to assay the *in vitro* transformation frequencies of *S. pneumoniae* isolates belonging to different serotypes. All the serotypes investigated had similar transformation frequencies, ranging from 2.6 × 10^-5^ to 7.9 × 10^-8^ transformants per recipient cell, with no significant difference between drug-resistance and drug-susceptibility associated serotypes (Joloba et al., 2010). Nowadays, next generation sequencing technologies allow the rapid and parallel sequencing of 100s of genomes and bring new knowledge on the evolution of *S. pneumoniae* genome. Many of these studies are aimed at finding recombination events due to transformation and therefore exclude the recombination events due to MGEs (Mostowy et al., 2014). The seminal study by Croucher et al. (2011) examined the evolution of ICESp23FST81 in the PMEN1 clone and found that the acquisition of macrolide resistance genes on Tn*916* occurred independently multiple times across the phylogeny. Another work was focused on PMEN2 and PMEN22 lineages, both carrying an independently acquired Tn*5253* family ICE. In some PMEN2 isolates from Iceland, recombination events lead to inactivation or loss of resistance determinants, interestingly one of these events was the precise excision of the Tn*916*-like element from the Tn*5253* backbone (Croucher et al., 2014). A recent sequencing study was performed on 3,085 nasopharyngeal pneumococcal isolates collected from infants and their mothers in a refugee camp over a period of 3 years (Chewapreecha et al., 2014). A total of 2,209 putative recombination events were detected among strains of different lineages, out of these 191 (8.5%) were plausible capsular switches while 132 (6%) were possibly due to MGEs. These data cannot confirm whether the transfer is due to conjugation or transformation, but underline how horizontal transfer of MGEs contributes to pneumococcal genome plasticity and likely utilizes at least two different mechanisms for horizontal gene transfer.

## EVIDENCE FOR Tn*916*-LIKE ELEMENTS IN MEMBERS OF THE ORAL STREPTOCOCCI – A DIVERSITY OF RESISTANCE GENES

Following the original description of Tn*916* (Franke and Clewell, 1981) reports of transferable tetracycline resistance in oral streptococci were published soon after. The host species of these Tn*916*-like elements were *S. mutans* U202 (Hartley et al., 1984), *S. anginosus* (Clermont and Horaud, 1990), and *S. mitis* (Bentorcha et al., 1992). The majority of these early studies were based on Southern blots being probed with wildtype Tn*916* sequences, however, these techniques, while confirming the presence of Tn*916*-like structures were not sophisticated enough to show all the details of the elements involved. Following the advent of modern day sequencing and improved PCR techniques it became possible to gain a better understanding of the genetic context of the resistances found in the oral streptococci. Two recent studies have focussed on characterizing the whole element responsible for tetracycline resistance in oral streptococci. In the first study (Ciric et al., 2012) the authors used a PCR based method followed by RFLP profiling of large amplicons spanning the entire length of Tn*916* (18,032 bp). They showed that wildtype Tn*916* elements were not the most common element found in the tetracycline resistant streptococci cultured from the saliva of healthy humans. Rather the majority of elements were Tn*6002* and Tn*3872* which were found in strains where these elements have never been reported before; Tn*3872* in *S. salivarius* and *S. sanguinis* and Tn*6002* in *S. australis, S. infantis, S. mitis, S. oralis, S. parasanguinis, S. salivarius*, and *S. sanguinis* (Ciric et al., 2012). In a larger, more recent study 263 resistant viridans group streptococci were analyzed in order to elucidate the genetic basis of resistance (Brenciani et al., 2014). The authors showed the presence of multiple new structures of MGEs based on Tn*916*-like elements and, similarly to both the previous study (Ciric et al., 2012), and the situation previously described for *S. pneumoniae* (see above) showed the co-carriage of multiple resistance genes, e.g., *tet*(M) and *erm*(B) on Tn*916*-like MGEs in different oral streptococci. The architecture of these different accessory elements responsible for incoming resistances has been described above and reviewed relatively recently (Ciric et al., 2011a; Roberts and Mullany, 2011).

This acquisition of multiple resistance genes by Tn*916*-like elements is an emerging theme in studies analysing the entire element present in the oral and nasopharyngeal streptococci, and the authors suggest that researchers analyse the entire element before reporting the presence of Tn*916* based simply on the detection or one or two genes by PCR, such as the integrase gene. In support of this suggestion it is also worth mentioning that the presence of *intTn* has been detected in excess of *tet*(M) in oral metagenomic DNA samples which strongly suggests there are elements containing the integrase gene which do not contain *tet*(M; Seville et al., 2009) and indeed a small number of these *tet*(M)-free Tn*916*-like elements have been reported from oral streptococci, e.g., Tn*916*S from *S. intermedius* (see below; Lancaster et al., 2004; Novais et al., 2012a).

## ANTISEPTIC RESISTANCE IS CO-LOCALIZED ON Tn*6087* WITH TETRACYCLINE RESISTANCE IN AN ORAL *S. oralis*

During the previously described survey of Tn*916*-like elements in oral tetracycline resistant streptococci (Ciric et al., 2012) a strain of *S. oralis* was isolated which contained a novel element; detected by showing a different RFLP pattern following digestion of the long amplicons derived from Tn*916*. Upon further analysis and subsequent sequencing the authors demonstrated the presence of additional DNA inserted within *orf15* of Tn*916* (Ciric et al., 2011b). Analysis of this region showed that there were two almost identical copies of IS*1216*, an insertion sequence which is commonly associated with *tet*(S) containing Tn*916*-like elements in the enterococci (Novais et al., 2012b). Between these two copies of IS*1216* there are two ORFs, one predicted to encode a hypothetical protein of unknown function and the other a small multi-drug resistance protein which was shown, by mutational analysis to encode resistance to cetyltrimethylammonium bromide (CTAB). CTAB is an antiseptic which is effective against bacteria and fungi and is one of the components in the topical antiseptic cetrimide used widely to treat minor skin injuries, minor burns, scalds, and nappy rash. In order to determine how this gene cassette can associate and insert within Tn*916*, PCRs were carried out across the region of insertion with primers designed to amplify a circular form of the small antiseptic resistance composite transposons and ligated target sites in Tn*916*. Multiple different products were isolated which showed that the small composite transposon consisting of the two IS elements and the intervening DNA could be detected within its target site, the antiseptic resistance gene and one copy of IS*1216* could be detected in a circular form, one copy of a chimeric IS*1216* could be detected in the target site within *orf15* and finally the restored target site was detected by PCR. These products suggest that there are multiple mobility mechanisms being utilized by this element, the activity of the IS*1216* encoded transposase could explain the excision reactions leading to the circular form detected, conversely homologous recombination between the two copies of the element must have led to the formation of the chimeric version of IS*1216* which was amplified. This redundancy in the mobility of the DNA gives a possible scenario for the continued isolation of novel variants of Tn*916*-like elements. Insertion into a replicon, due to homologous recombination presumably negates the need for transposition reactions to occur in order to obtain successful integration into the Tn*916* backbone. An interesting example of a recombination within Tn*916* is seen within the conjugative transposon Tn*916*S from an oral *S. intermedius*. It was originally (and erroneously) described as a Tn*916*-like element containing the *tet*(S) gene in place of *tet*(M) (Lancaster et al., 2004). More recent analysis of the sequence of this element, however, has shown that it is infact a hybrid element containing a mosaic tetracycline resistance gene composed of *tet*(S) (encoding 599 amino acids of the gene) from a related element from *Enterococcus casseliflavus* designated Tn*6000* (Roberts et al., 2006; Brouwer et al., 2010) and the Tn*916* wildtype *tet*(M) gene (encoding 61 amino acids of the hybrid gene) and designated *tet*(S/M) (GenBank accession no. AY534326.1; Novais et al., 2012a).

Interestingly with Tn*6087* the target site for the small composite transposon encoding *qrg* was within *orf15*. Despite repeated attempts the authors could not detect transfer of tetracycline and CTAB resistance by conjugation, however, they did show transformation into an oral *S. australis* (Ciric et al., 2011b).

## CONCLUSION

The repeatedly observed redundancy in transfer mechanisms (conjugation and transformation) of many of the Tn*916*-like elements described in this review will likely lead to a higher chance of successful transfer than if these elements only relied on conjugation in order to spread. This may be one of the reasons why the Tn*916*-like elements are so successful within this genera and why there is so much variation of these elements in oral and nasopharyngeal streptococci. The elements themselves; both Tn*916* and the smaller elements found within it, may well have exploited the competence inherent in many species within this genera.

## Conflict of Interest Statement

The authors declare that the research was conducted in the absence of any commercial or financial relationships that could be construed as a potential conflict of interest.
